# The first family of application-specific integrated circuits for programmable and reconfigurable metasurfaces

**DOI:** 10.1038/s41598-022-09772-y

**Published:** 2022-04-06

**Authors:** Loukas Petrou, Kypros M. Kossifos, Marco A. Antoniades, Julius Georgiou

**Affiliations:** 1 Department of Electrical and Computer Engineering, University of Cyprus, Nicosia, Cyprus; 2 Department of Electrical, Computer and Biomedical Engineering, Ryerson University, Toronto, Canada

**Keywords:** Electrical and electronic engineering, Electronic and spintronic devices

## Abstract

Reconfigurable metasurfaces are man-made surfaces, which consist of sub-wavelength periodic elements—meta-atoms—that can be reconfigured to manipulate incoming electromagnetic waves. However, reconfigurable metasurfaces developed to-date, have limitations in terms of loading impedance range, reconfiguration delay and power consumption. Also, these systems are costly and they require bulky electronics and complex control circuits, which makes them unattractive for commercial use. Here, we report the first family of CMOS application-specific integrated circuits that enable microsecond and microwatt reconfiguration of complex impedances at microwave frequencies. Our approach utilizes asynchronous digital control circuitry with chip-to-chip communication capabilities, allowing simple and fast reconfiguration via digital devices and user-friendly software. Our solution is low-cost and can cover arbitrary board-to-board metasurfaces, with different sizes and shapes.

## Introduction

Metasurfaces are electrically thin composite materials that consist of periodically spaced, sub-wavelength meta-atoms. These composite materials have gained the interest of many researchers, due to their ability to demonstrate novel functionalities, such as perfect absorption^1–6^, anomalous reflection^7–9^, beam shaping^10^, and more.

Reconfigurable metasurfaces have also been demonstrated by incorporating various tunable elements within the meta-atom. Electrical tunability has been obtained using varactor diodes^11,12^, complex-impedance loading elements^3–6^, liquid crystals^13^, transistors^14^ and amplifiers^15^. Besides electrical tunability, other means of tunability have been demonstrated, such as magnetic^16^ and optical^17^ tunability. Optically tunable behavior has also been shown by utilizing the optical properties of PDR1A^18^, which has been shown to possess a memory effect^19^.

Recently, programmable metasurfaces have emerged^15,20–27^. These are tunable metasurfaces that control individual meta-atom states, through software. Often, the control of each meta-atom is binary and is implemented through a field-programmable gate array (FPGA) development board^20,22,26^. An increase in meta-atom states was achieved by incorporating digital-to-analog converters between the FPGA and the tunable elements of the meta-atom^15,21,23,24^. With the individual meta-atom control, programmable metasurfaces have demonstrated multifunctional applications e.g. polarization, scattering and focusing control^20^, multi-focal spot control^21^, holography^22^, imaging^24^, non-linear harmonic manipulation^23^ and even beam steering while controlling the harmonic power level^25^. The advanced electromagnetic manipulation that programmable metasurfaces possess has made them an attractive solution for future wireless telecommunications. A recent study^28^ showed that improved wireless connectivity can be achieved by incorporating metasurfaces in telecommunication systems. Even though this technology is still in its infancy, basic experimental verifications of simplified telecommunication systems based on programmable metasurfaces were shown^29,30^.

Although the incorporation of programmable metasurfaces in wireless telecommunication systems can provide a clear advantage over traditional MIMO implementations and transceiver architectures^29,30^, there are major drawbacks that need to be addressed before they can be adopted. In particular, such programmable metasurfaces need to be cost-effective, scalable, low-power and low-noise. The solution with FPGAs feeding discrete shift registers, to drive multiple digital-to-analog converters, that finally bias multiple power-hungry varactor diodes, although promising, cannot address the requirements for real-time programmability of metasurfaces. Furthermore, the bulky and complex electronics required in these systems limits their expandability and the option to be used by engineers that are not familiar with that particular system.

To address all of these requirements, major upgrades in the programmable metasurface architecture need to be implemented. In this paper, we present the first family of application-specific integrated circuits (ASICs) designed for programmable metasurfaces, which satisfy all these requirements. The ASICs can become part of a metasurface by connecting and controlling the surface impedance of each meta-atom via software. They incorporate in a single die an asynchronous control circuit, multiple digital-to-analog converters and multiple complex impedance tunable elements.

## Metasurface ASIC characteristics

The ASICs utilize an asynchronous methodology for the control circuit to reduce noise and increase speed, while simultaneously consuming less power^31^, when compared to traditional clocked systems. The digital-to-analog converters’ eight-bit resolution provides fine adjustment of the low-power complex impedance loading elements^3^. Four complex impedance loading elements are implemented in each ASIC, and can be individually addressed to provide more flexible control over the metasurface meta-atoms. As was shown in simulations^3^ for preliminary designs, we are able to show control of amplitude and phase of the reflection coefficient for both polarizations, independently and simultaneously. This ability was exploited to not only perfectly absorb an incident wave, but also to manipulate and transform various wavefronts^32–34^. In this work, we are able to control the instantaneous amplitude and phase response of the meta-atom, in real time, using low-noise and low-power, asynchronous chips. In addition, integrating all the electronic components into one package reduces the cost and size, and increases their performance, making them suitable for programmable metasurface designs which require a large number of electronic elements.

## Circuit architecture and implementation

**Figure 1:**
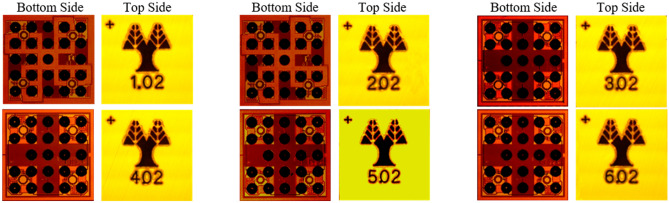
Microphotographs of the ASIC chip family for programmable metasurfaces, showing both top and bottom sides. The ASICs use Wafer Level Chip Scale Package (WLCSP)^35^ technology, where the solder-bumps (seen on the bottom side) are directly placed on the I/O pads of a redistribution layer beneath the wafer.

The manufactured ASICs are shown in Fig. 1. The ASIC family is formed by six similar mixed-signal designs. Each ASIC version is optimized for a nominal center frequency and with a different tunable impedance range to provide additional flexibility, to cater for various metasurface designs. The chips’ footprint and pin allocation can be found in the Supplementary Information Note 1. At the top side of the chips, the numbers indicate the design (1–6) and the wafer number (01–12). Figure 1 shows chips from wafer number 02.

**Figure 2:**
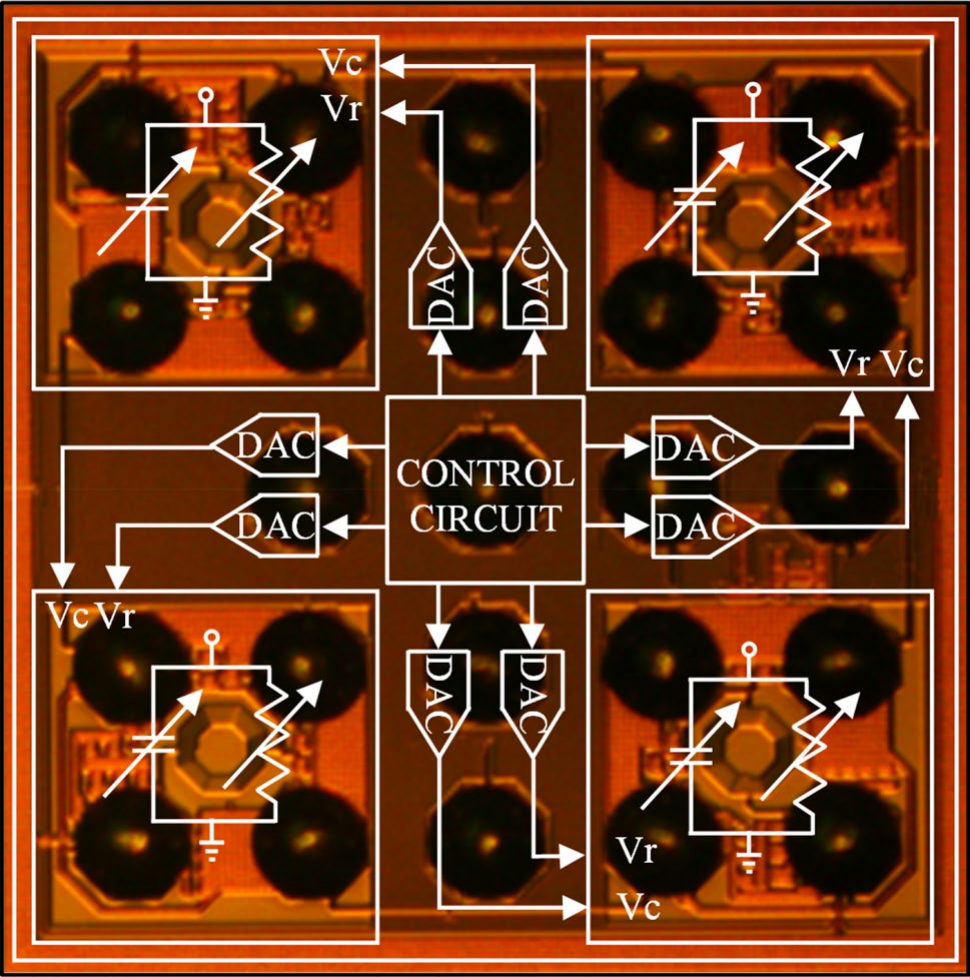
Microphotograph of the bottom side of ASIC Design 4.02 with the overlaid architecture. Large top-metal structures of the chip, such as inductors, can be seen between solder-bumps. The digital control circuitry is hidden behind metal-shielding layers.

The internal architecture of the foundational ASIC is overlaid on the physical layout in Fig. 2, to illustrate the correlation between the physical layout and the architecture. The architecture consists of four loading elements, eight digital-to-analog converters and a control circuit. Each loading element utilizes a MOSFET varistor and a MOSFET varactor to adjust the real and imaginary part of the impedance, respectively, while simultaneously having negligible static power consumption since their consumption consist of the leakage current through eight RF MOSFETs which is very low compared to the rest of the circuits. Eight, 8-bit digital-to-analog converters are used to finely tune the varistor and varactor of each loading element of the ASIC. The digital inputs of the digital-to-analog converters are provided by the digital control circuit located at the center of the chip. The control circuit has two main operations. The first is to be part of a communication grid that will send/receive data packets to/from neighboring nodes, in order to store the appropriate payload in its memory. The second is to provide the necessary digital inputs to the digital-to-analog converters, and in turn to tune the surface impedance of the corresponding meta-atom.

The configuration of the chip can be achieved using an external digital device e.g. FPGA or microprocessor. However, the digital device can be either removed or enter sleep mode once the configuration of the chip/s is completed, since the data are stored in the memory cells of the chips. Thus, the power consumption of the digital device is not considered part of the static consumption of a programmable metasurface system that utilizes the proposed chips. Each chip can also communicate and exchange packets with other chips from this chip-family. This way, we can form an array of chips with serial communication capabilities embedded in the metasurface structure. Further extending this idea, we can have one chip per meta-atom of a metasurface and be able to programmatically adjust its impedance via software. With our low-power design, we can power metasurfaces of a few square meters using only batteries, which may be recharged using energy scavenging methods^36^. Also, the chip is a standalone device i.e. there is no need for any other external component on the metasurface, thus keeping the cost to a minimum. Furthermore, a metasurface system can be designed without any external cables, bulky electronics, external components etc. Although the idea is simple and effective, there are numerous constraints imposed, due to the implementation at microwave frequencies and the limitation from state-of-the-art integrated circuit manufacturing processes. These constraints are present for all programmable metasurface systems utilizing integrated circuits.

The ASICs were implemented in a mainstream CMOS 0.18 µm semiconductor technology that balanced cost per die, mixed-signal capabilities, RF characteristics as well as low-power digital circuit capability operating at 1.8 V. Our proposed chips can realize reconfigurable metasurfaces and adjust the complex loading impedance of each meta-atom individually. Depending on the application and metasurface design, a large number of chips are required^4^. These could amount to thousands of chips for a one square-meter metasurface, thus chip cost is a critical factor. Wafer Level Chip Scale Packaging (WLCSP) technology was selected because it enables both better RF performance, since bond wire parasitics and variations are minimized, as well as lower cost per chip when fabricated in large quantities because the process of wire-bonding is not required and also the solder-balling process is much simpler and cost effective. The diameter of the solder-ball spheres and the pitch of the balls were chosen to be 250 µm and 400 µm, respectively. Smaller solder spheres and smaller pitch sizes are available, but this increases the printed circuit board (PCB) costs, on which the metasurface is patterned. For smaller pitch sizes, special PCB processes are required and the reliability decreases. Therefore, the balls and pitch were kept at a size to comply with widely available high-frequency laminate PCB manufacturing design rules. The size of the ASICs was chosen to be 2.2 mm × 2.2 mm to increase their yield and subsequently keep the cost relatively low. The size of the chips and pin pitch subsequently dictated the number of available input/output (I/O) pins to be twenty-five. The size of the ASIC and the pin limitation add constraints to the chip architecture. The number of available loading elements were chosen to be four and this dictates the number of digital-to-analog converters, and subsequently the control circuit’s complexity, communication, and control scheme. Due to the pin limitation, we were forced to adopt a serial communication scheme between the chips.

The control circuit uses asynchronous circuits and communicates via handshake. Synchronous digital circuits are by far predominant in control circuit designs, however to maximize the ASIC usability for various metasurface designs, and to avoid all issues associated with the clock tree synthesis of synchronous digital systems, we opted to go the asynchronous route^31^. A synchronous digital system would require a clock tree with scalability adjustments and would have to satisfy the requirements of flexible metasurfaces and walls of irregular shapes. In addition, the clock skew would have to be well controlled to eliminate any setup and hold time violations. Addressing all these with a synchronous digital system, would require a static system with scalability limitations. By using asynchronous digital circuits, the scalability became as simple as connecting meta-atoms or tiles of metasurfaces together, since there is no clock signal to synchronize the operations. Once the wires are connected, the system can operate without requiring any modifications or optimizations. Power consumption is also minimized by using the asynchronous circuit approach. At static conditions, the asynchronous control circuit consumes only leakage current whilst the synchronous counterpart would consume both static and dynamic current, at every clock cycle. Specialized circuitry and techniques can be adopted to turn off certain parts of the chip when not needed, but still the buffers of the clock tree would be enabled. Also, the available area for the control circuit is limited due to the small size of the chip and such techniques would consume a significant amount of our free space allocated for the digital-to-analog converters and the loading elements. Another major advantage from the asynchronous circuit approach, is related to the significantly reduced levels of electromagnetic emissions that are generated during programming^37^. A synchronous approach generates significant amounts of broadband noise during switching activity, given all transitions will happen at the same time. This creates problems for metasurface absorber^3–5^ applications, given that the surface will be radiating EM waves at each clock event. This is not the case in our control circuit because the noise generated is lower and more evenly spread timewise, since only the chips that exchange data are enabled, while the rest are idle.

## Experimental results

The performance and properties of the ASICs are divided in two parts since the RF circuits and asynchronous digital circuits require different test-setups and experiments to extract their performances. We first describe the performance achieved from the RF loading elements and then we describe the performance achieved from the asynchronous digital control circuit and the digital-to-analog converters.

### RF characteristics

**Figure 3:**
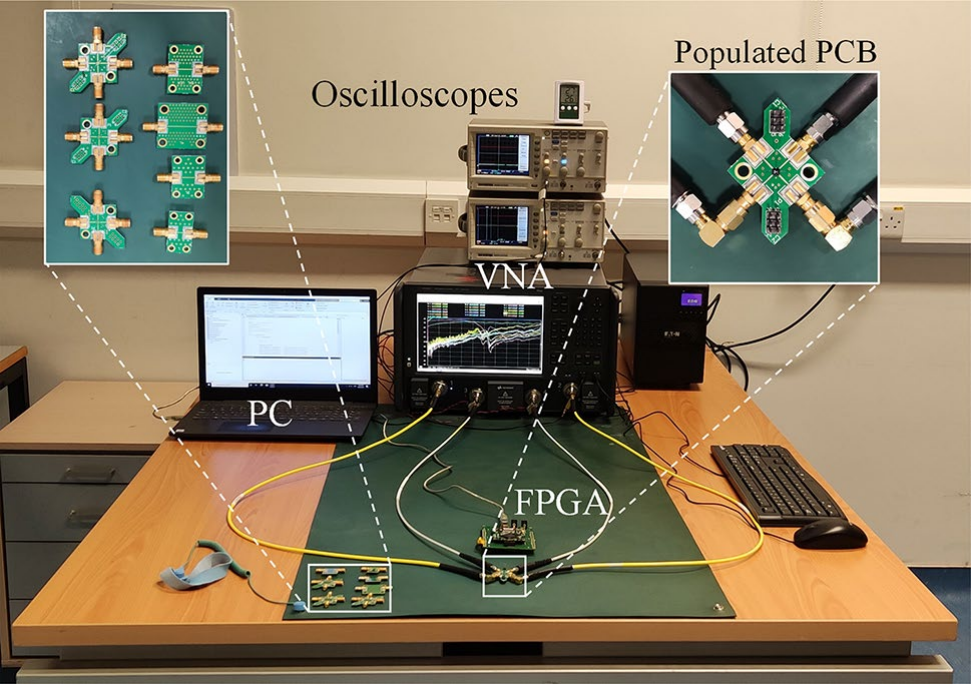
Loading element measurement setup. The PC on the left side of the image, calculates and sends the configuration settings to the populated PCB, which includes the ASIC under test, through an FPGA interface board. To extract the RF characteristics of the chip, as shown on the populated board in the top right inset, the de-embedding boards, shown in the top left inset, are used to characterize and eliminate the effects of all lines leading up to the chip’s solder bumps. The S-parameters are captured on the 4-port Vector Network Analyzer (VNA) and afterwards the real and imaginary part of the impedance is calculated.

**Figure 4:**
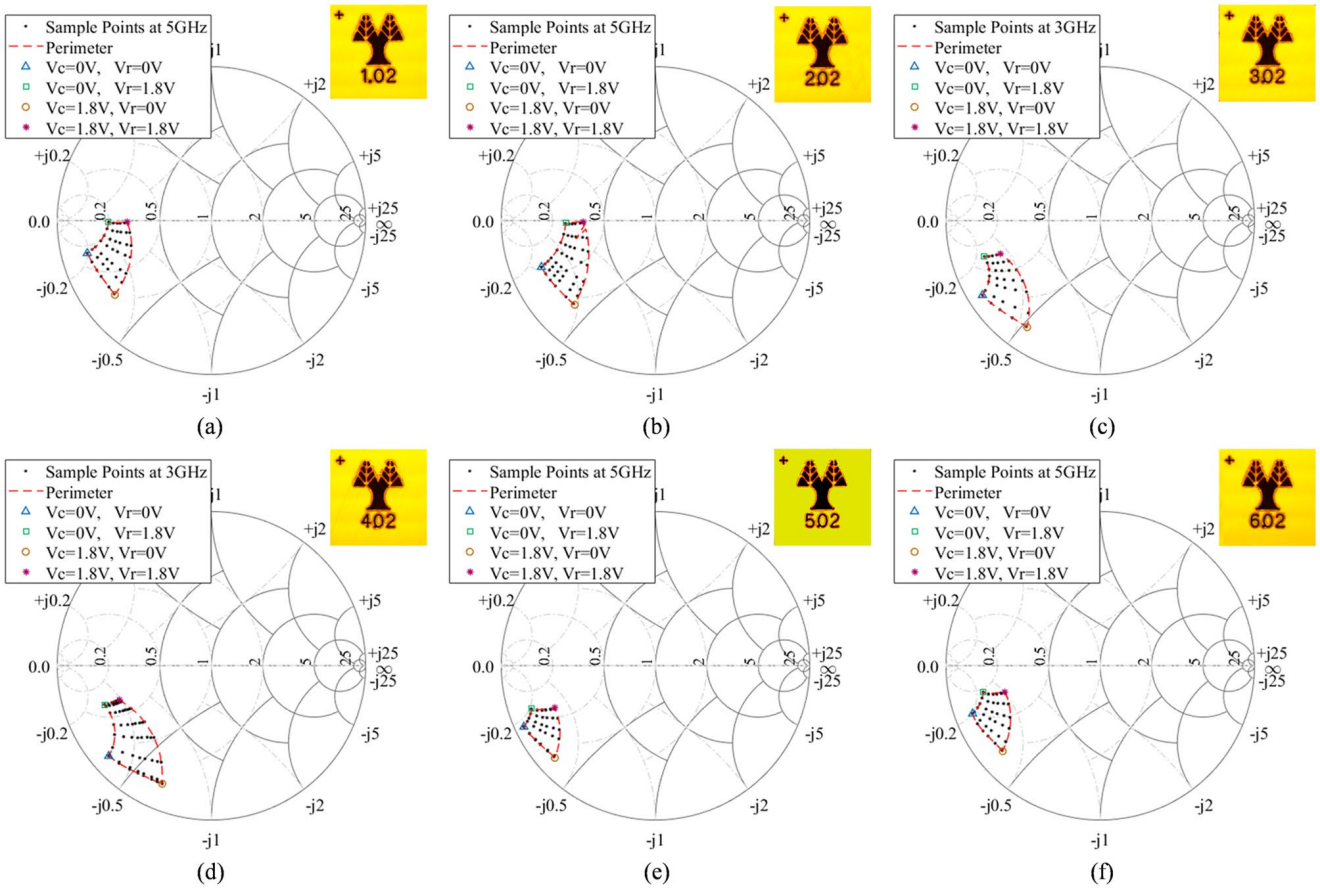
Smith Charts illustrating the de-embedded measured impedance responses of the loading elements for each of the six ASIC designs. The different ranges of impedances for each ASIC can be observed for bias voltages ranging from 0 < Vc < 1.8 V and 0 < Vr < 1.8 V. The red dashed line delineates the perimeter of the measured impedances at 5 GHz for all bias levels, indicating wide and varying realized impedance ranges.

**Figure 5:**
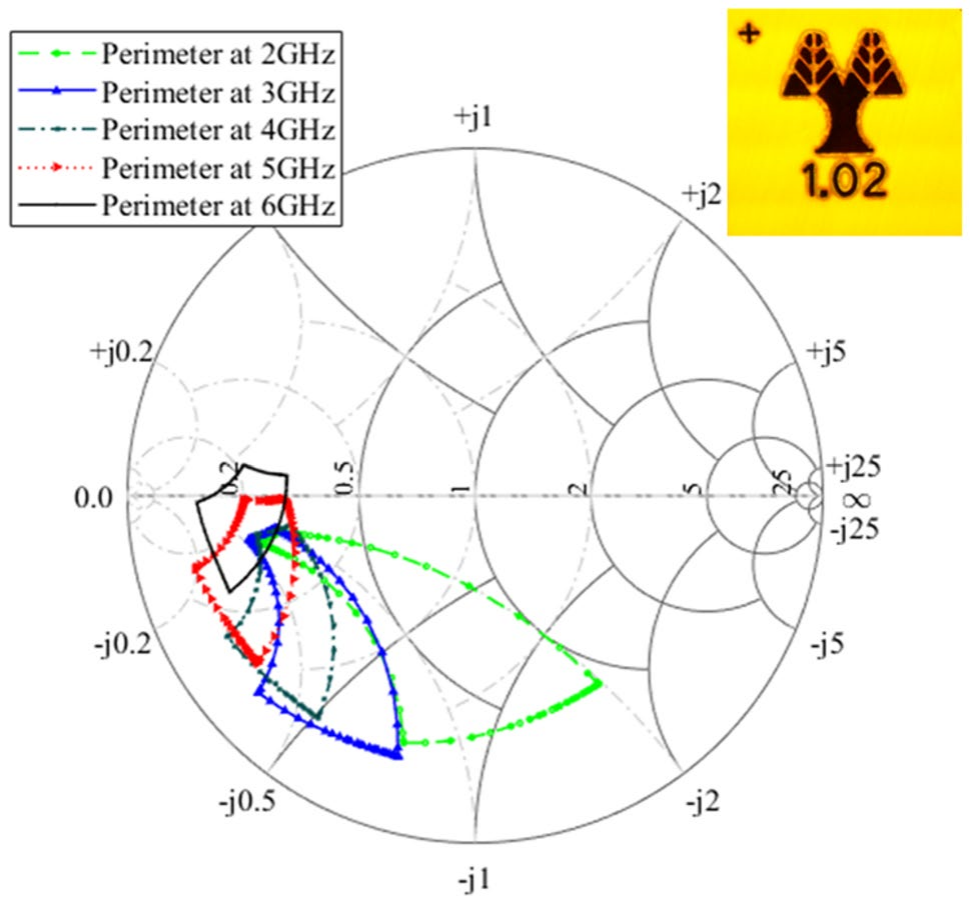
Smith chart illustrating the de-embedded measured loading element impedance perimeters at various frequencies of ASIC Design 1.

The performance of the RF loading elements was evaluated with the test-setup shown in Fig. 3 and the results are presented in Figs. 4 and 5. The loading element design methodology was reported in an earlier work^3^, but these measurements were taken by directly probing the structures on-chip, and do not include the parasitics of routing to the chip corners as well as the WLCSP parasitics. Furthermore, the previously presented measurements used external supplies to bias the loading elements whereas in Figs. 4 and 5 the adaptive bias is generated from on-chip DACs, with no external supply. The resistance and capacitance values of the loading elements are tuned with the DAC output voltages Vr (real impedance bias) and Vc (imaginary impedance bias), respectively, as shown in the ASIC architecture of Fig. 2. The measured impedance responses of the loading elements for the entire family of chips are shown in Fig. 4 on Smith charts. Note that each chip was optimized for a different operating regime. The designs have a wide operating frequency from 2–6 GHz, and in this work, we show representative results for five frequencies within this frequency range. The frequency dependency is illustrated in Fig. 5 for Design 1 by showing the perimeter at 2, 3, 4, 5 and 6 GHz. Designs 1,2,5 and 6 have a center frequency of 5 GHz, while Designs 3 and 4 have a center frequency of 3 GHz. At the center frequency, an area on the Smith chart is outlined, where the loading element can be operated. This area is formed by controlling the input code of the two digital-to-analog converters that bias the loading element at the Vr and Vc nodes. The measured perimeter is plotted with a dashed red line at the center frequency. Sample points are also plotted at the center frequency to illustrate the ability to obtain intermediate states in the plotted area.

### Digital control circuitry characteristics

**Figure 6:**
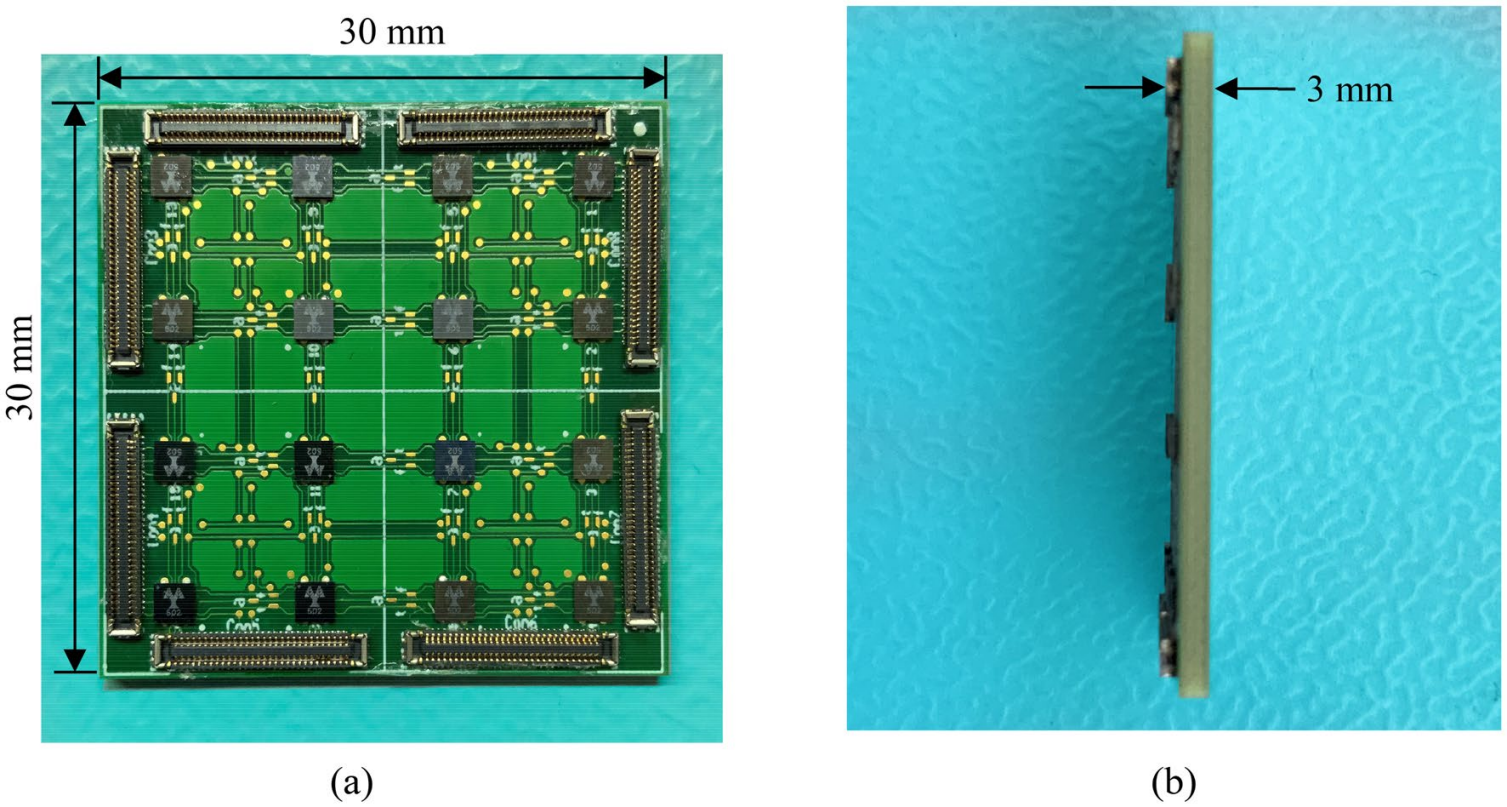
Photograph of a 4 × 4 chip grid. (**a**) Front view, and (**b**) side view. Note the very small thickness of the populated test-board.

In order to extract the properties of the asynchronous digital control circuit, we fabricated a test-board consisting of 16 ASICs in a 4 × 4 grid configuration as shown in Fig. 6. This test-board does not include the metasurface part of the system i.e. the metal-patches at the front side of the PCB and the high-dielectric PCB layers. However, it has a similar design and identical routing layers with the envisioned programmable metasurface. This way, the test-board provides information regarding the delay, scalability and energy consumption of the ASICs while operating in a similar environment to that of a programmable metasurface. The size of the test-board is 30 mm × 30 mm as shown in Fig. 6a while the thickness of the test-board is just 3 mm including the chips and the connectors, as shown in Fig. 6b. The chips can be also attached on flexible surfaces and form irregular shapes since as mentioned, the asynchronous circuit design adopted does not depend on the delay of the wires and MOSFET gates for valid communication, unlike a synchronous circuit design.

**Figure 7:**
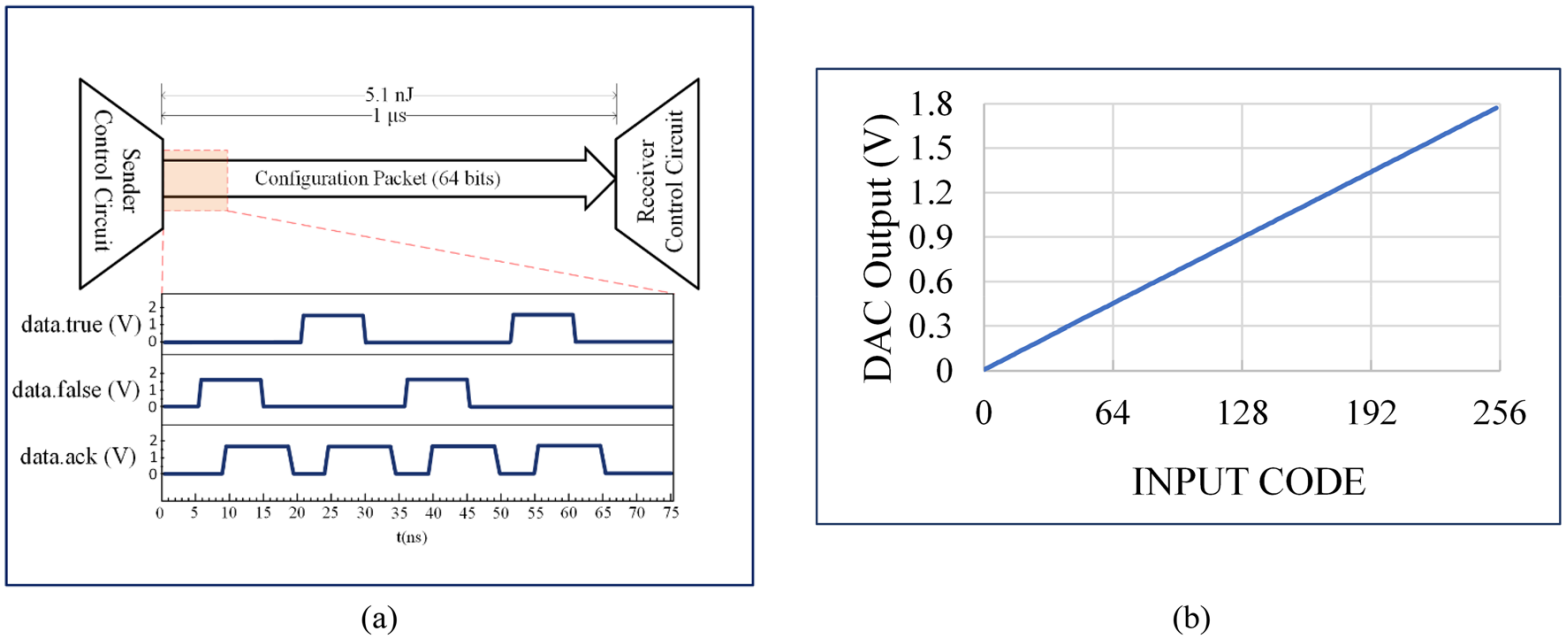
Controller measurements, obtained using an FPGA controlled by a PC, while the test-board was powered using a source-measure-unit (SMU) device. (**a**) Voltage waveforms of the three data signals (data.true, data.false and data.ack) generated by the asynchronous digital control circuit for the binary sequence ‘0101’, together with an illustration of the data flow between neighboring chips in a typical serial communication scheme and (**b**) output voltage of the ‘Digital-to-Analog Converter (DAC) as a function of the input code.

The test-setup consists of a PC and an FPGA to prepare and send the data-packets to the chips, and an oscilloscope to visualize the results. The chips are powered using a reliable source-measure-unit (SMU) device, to facilitate average power consumption measurements. Each control circuit has sixty-four memory cells connected in series configuration, and has eight cells allocated per digital-to-analog converter. Thus, the packet length is sixty-four bits and the total number of individual states available per chip is 2^64^. Neighboring chips (sender and receiver in Fig. 7a) communicate via handshake and respect the 4-phase dual-rail asynchronous protocol, whereby each bit is represented by two signals (‘data.true’ and ‘data.false’) and there is also the acknowledge signal (‘data.ack’) which declares the end of each cycle. A detailed explanation of the 4-phase dual-rail protocol can be found in the Supplementary Information Note 2. Due to the 4-phase dual-rail asynchronous nature of the circuits, the ASIC’s configuration time is not dependent on the data packet content i.e. the cycle time is independent of the binary sequence transmitted or received. The average configuration time per chip is measured to be 1 µs. During this time, in the channel, we exchange 64 bits between the sender circuit and the receiver circuit and both circuits are ready for the next packet. To exchange one bit between two ASICs, the average required time is 14 ns, thus the bit rate of the ASIC is 68 MBits/s. The static power consumption of the ASIC is 328 µW. The energy per bit consumed by one ASIC is 79 pJ/bit and the energy consumed per ASIC reconfiguration is 5.1 nJ/packet considering the delays of the buffer stages and the PCB tracks between the chips. Note that the power consumption could have been less but the ASIC design consists of four impedance loading elements with eight digital-to-analog converters (main power contributors) and thus it can set the impedance of up to four meta-atoms. Figure 7a also shows the measured signal transition for the binary sequence ‘0101’ which is sent from the sender ASIC to the receiver ASIC respecting the asynchronous protocol. The data rises first (‘data.true’ or ‘data.false’ = ‘1’), followed by a rise in the acknowledgment (‘data.ack’ = ‘1’) after the data has been stored at the receiver’s memory cell. To end the cycle, the data return to its starting state (‘0’) followed by the acknowledge returning to its starting state (‘0’). These results imply that a configured metasurface consisting of a few thousand meta-atoms with one chip per meta-atom, would consume power on the order of a few milliwatts, easily provided by small-sized batteries or through energy-harvesting means. The reconfiguration is performed with a user-friendly software, which is described in Supplementary Information Note 3. Furthermore, in a few milliseconds, we can change the configuration data of each meta-atom individually and apply one of the 2^16^ values for each of the four available loading elements per chip. Our digital-to-analog converter design is based on a two-stage resistor string architecture, with a resolution of eight bits. The advantages of this digital-to-analog converter type are its accuracy, monotonicity, and its small layout. Also, it can be directly connected to the loading elements since its output impedance is much lower than the controlling input impedance of the varistors and varactors. Figure 7b shows the output voltage for each of the 256 binary inputs. The ASIC’s measured performance and characteristics are summarized in Table 1. The static power consumption is mainly attributed to the digital-to-analog converters, whereas the dynamic consumption is dependent on the reconfiguration frequency, hence characterised as energy consumption per packet.

**Table 1:** Characteristics for the proposed metasurface ASICs.

Technology	0.18 µm CMOS mixed-signal RF 1P6M
Supply voltage (core and IO)	1.8 V
Die size	4.84 mm^2^
Package type	WLCSP
Number of memory cells	64
Bit rate	68 MBits/s
Energy consumption per bit	79 pJ/bit
Packet frequency	1 MHz
Energy consumption per packet	5.1 nJ/packet
Static power consumption	328 µW
Digital-to-analog converter range	0–1.793 V
Digital-to-analog converter resolution	7 mV/bit

### Reconfigurable metasurface design approach comparison

In Table 2 we compare various programmable MSF design approaches, where the strengths and weaknesses of their enabling mechanisms are outlined. We divide the designs into discrete component-based designs and ASIC-based designs. Although the comparison is by no means exhaustive, it gives the reader insight on the benefits offered by each approach and the challenges that are faced in creating similar systems.

**Table 2:** Comparison with other tunable and programmable metasurface enabling circuits indicating the strengths and weaknesses of all approaches to give the reader insight of the best approach suited for various applications.

	Discrete component-based designs						ASIC-based designs			
	Ref.^20^		Ref.^38^		Ref.^39^		Ref.^27^	This work		
Principle Components	PCB + PIN Diodes + FPGA		PCB + PIN Diodes + control station		PCB + Varactor Diodes + external tuning voltage		MOS switch-based, variable split ring resonator	PCB + ASIC varactor/varistor based loading elements with communication circuitry		
Controller Type	Centralized FPGA		Centralized Control Station		Centralized Biasing Voltage Source		Centralized Synchronous Digital	De-centralized Asynchronous Digital		
Individual Control of Meta-Atoms	Yes		Yes		No (Group Control)		Yes	Yes		
States per Meta-Atom	2		2		6		2^8^	1 LE	2 LE	4 LE
								2^16^	2^32^	2^64^
Operating Frequency	11 GHz		5.8 GHz		10 GHz		0.3 THz	2–6 GHz		
Operating Voltage	1.33 V		1.33 V		8 – 13 V		1.2 V	1.8 V		
Support Multiple metasurfaces with low incremental cost	Yes		Yes		Yes		No	Yes		
Expansion Capabilities	Yes (FPGA re-cabling and re-coding)		Yes (Control Board expansion)		Yes (Increase Number of Tuning Voltages)		Yes (PCB re-design)	Yes (Plug-and-Play, no re-design)		
Scalability	High		High		High		Medium	Very High		
Static Power Consumption per meta-atom	ON	OFF	ON	OFF	ON	OFF	1.67 µW	1 LE	2 LE	4 LE
	100 mW	Negligible	100 mW	Negligible	100 mW	Negligible		82 µW	164 μW	328 µW
ASIC to Meta-atom coverage ratio*(CMCR)	N/A (not ASIC based)		N/A (not ASIC based)		N/A (not ASIC based)		1	0.07		

*CMCR is defined in (1).

The discrete component-based approaches (Refs.^20,38,39^) aim for simple hardware, by having the intelligence of the system in the controller system. They consist of a PCB metasurface divided into meta-atoms, with each meta-atom having a discrete diode component to control its surface impedance. Furthermore, all approaches compared, use a centralized controller to control the states of all principle components individually, or in groups. Specifically, Ref.^20^ uses an FGPA device to individually set a binary state to each PIN diode. Ref.^38^ uses a control station that includes a control PCB, a supply PCB, a power supply unit and a laptop to individually set a binary state to each PIN diode. Ref.^39^ uses biasing voltages from a centralized voltage source, with each voltage biasing an array of 2856 varactor diodes. The voltage varies from 8 to 13 V with a 1 V increment, thus the available states in this design are six. Most of the discrete component-based approaches operate in the 5 to 60 GHz range, where many of today’s communication systems operate. One of the main strengths of these systems, is the simplicity of the metasurface hardware, which allows support of multiple metasurface designs with a relatively low incremental cost, since the diodes can be easily soldered on various boards and controlled by the centralized unit. In addition, these systems have expansion capabilities by simply connecting boards to the centralized unit, although more cables must be used in the system, which leads to bulky electronic systems. In the case of the FPGA controller (Ref.^20^), as the system is scaled up in size, the programming code needs to be adjusted accordingly to control the increased number of meta-atoms.. In general, the discrete component-based systems have a high scalability, although their power consumption is a critical limiting factor. This is because the power consumption of these systems can increase rapidly when the systems scale up. The power consumption depends on whether the principle component is ON or OFF. In the OFF state the current drawn by the diodes is negligible. However, during the ON state, the diodes consume 100 mW, which is a relatively high consumption, especially for a scalable system that includes a large number of diodes.

On the other hand, the ASIC-based approaches target low-power systems with many programmable states per meta-atom. They can have a centralized (Ref.^27^) or a decentralized (our work) control of their meta-atoms. Ref.^27^ uses a synchronous digital integrated control circuit for binary controlling eight MOSFET switches per meta-atom, allowing for 2^8^ states per meta-atom. The MOSFET switches form a variable split-ring resonator which produces the desired electromagnetic performance. This approach targets a very high operation frequency of 0.3 THz. This ASIC design cannot support multiple metasurfaces, since the ASIC has the metasurface fabricated on its top metal layer, which cannot change after fabrication. The system has expansion capabilities by connecting multiple ASICs on a PCB, although careful design of the PCB must be ensured, since the ASICs are very small (2 mm × 2 mm) and the wire-bonding procedure is complex in this situation. For these reasons, we consider the scalability of this design to be medium. The ASIC consumes very low power compared to the discrete component-based designs. The total current drawn by the ASIC at the 5 GHz switching frequency is given as 6.25 mA.

Our ASIC approach uses an asynchronous control circuit with communication capabilities and digital-to-analog converters to control the analog voltage of integrated MOSFET varistors and MOSFET varactors. The ASIC consists of four impedance elements offering up to 2^64^ states per ASIC. The design targets communication frequencies in the 2–6 GHz range. This ASIC approach is designed with cost and mass production in mind, aiming for an affordable, robust system with extreme scalability capabilities. The WLCSP technology along with the direct population of the ASICs on the metasurface offer a relatively low-cost solution with reliable manufacturing, that does not require any wire bonding. The idea is to form tiles of metasurfaces, e.g. each one with a 50 × 50 meta-atom array and edge connectors on each side to allow for expansion by simply connecting the tile-boards together. Our approach can be easily used to support other metasurface designs. This can be achieved by including a routing layer on the PCB for the ASICs to communicate, and through-via connections to connect the metallic patches to the loading element ports of the ASICs. We consider this system to be highly scalable, since the ASICs can communicate on multiple metasurface designs, both on rigid and on flexible structures, and allow for simple, plug-and-play expansion of the metasurface without any re-design. The scalability adjustments in conjunction with the relatively low power consumption enable metasurfaces with thousands of chips to be realized, covering walls of even up to few square-meters while consuming only milliwatts of power. Furthermore, the ASICs are very thin and with no exposed wires, thus the metasurface system is also thin and without excessive cables and bulky electronics (Fig. 6). The static power consumption is in the microwatt range, just like Ref.^27^. The slightly increased value of the power consumption in our case, comes from the fact that each ASIC has four loading elements and eight digital-to-analog converters, thus each chip can be used to control up to four meta-atoms. The largest amount of power is consumed by the digital-to-analog converters.

As a last metric, applied only to the ASIC based approaches, we define a figure of merit, the Chip to Meta-atom Coverage Ratio (CMCR), which relates the relatively expensive chip area to the cheaper meta-atom area, as follows:

1 $${\text{CMCR}} = {\raise0.7ex\hbox{${{\text{chip }}\;{\text{area}}}$} \!\mathord{\left/ {\vphantom {{{\text{chip }}\;{\text{area}}} {{\text{meta-atom }}\;{\text{area}}}}}\right.\kern-\nulldelimiterspace} \!\lower0.7ex\hbox{${{\text{meta-atom}}\;{\text{area}}}$}}$$

Equation (1) ranges between zero and one, where one gives the costliest solution for large-area adaptive metasurfaces because the overall cost of the meta-atom increases as the surface area of the chip increases. The CMCR reaches its upper limit at high frequencies, where the wavelength and thus the size of the meta-atom becomes so small that the CMCR eventually becomes equal to one, meaning that the back of the metasurface is covered entirely with chips. Of course, the PCB and chip manufacturers have limits on the maximum and minimum size of their fabricated boards and chips, thus to cover large surfaces beyond the manufacturing size limits, the designed system requires board-to-board connections and communication.

The conclusion that can be extracted from this comparison is that there is not an approach which is dominant, but rather all approaches have their strengths and weaknesses. The design choice is dictated by the intended metasurface application. The discrete component-based designs have limited states per meta-atom and consume relatively high power but they offer a simple hardware design, highly scalable systems at high frequencies with individual control of meta-atoms. On the other hand, the ASIC-based approaches offer many more states per meta-atom and consume much lower power. Depending on the design, the ASIC-based designs can target much higher operation frequencies (Ref.^27^), or focus on high expansion capabilities with low incremental cost (our work).

## Discussion

The presented family of ASICs is expected to drive a new generation of programmable metasurfaces that can be incorporated into future telecommunications systems, but which can also be retrofitted into current systems. In the programmable telecommunication environment presented in Ref.^28^, metasurfaces are used to improve the throughput and the security of indoor telecommunication systems. Outdoor telecommunication systems can be improved in multi-user environments by incorporating metasurfaces within base stations, thus improving their adaptive multibeam capabilities. The family of ASICs presented in this work is a key component in future metasurface-enabled telecommunications systems. Multifunctional performance has been demonstrated by incorporating the proposed ASICs in meta-atom structures illustrating their performance in combination with various metasurfaces^3–6^. With the advanced capability of tuning both the amplitude and phase of the reflection coefficient, the representative designs are able to perfectly absorb an incident wave up to oblique angles of incidence for both transverse electric and transverse magnetic polarizations. This has been further extended^3^ to absorb both polarizations simultaneously and independently. Anomalous reflection and polarization rotation were also demonstrated^5,6,39^, but even as is, these functionalities are only a fraction of what the ASIC-equipped programmable metasurface can achieve. By controlling the amplitude and phase of each meta-atom at a sub- wavelength scale, more complex functions of the metasurface design can be achieved, such as multi-beam patterns, absorption from one direction while reflecting in another, fine control of polarization, and non-linear reflection. For example, multi-beam or even arbitrary beam shaping to suit the needs of a telecommunication system in a multi-user and multi-objective environment could be achieved. Also, the advanced functionalities of such metasurfaces can be adopted in reduced radar-cross-section (RCS) applications, stealth technology, and even cloaking. Furthermore, programmable metasurfaces find applications in holography, which use the amplitude and phase programmability which they can provide.

The functionalities described in the literature, control the metasurface in two dimensions, engaging only amplitude and phase control with a slow or static operation. The family of ASICs presented herein utilizes fast asynchronous control circuits, which is also robust and reliable. Thus, the programmable metasurfaces that use these ASICs can quickly and accurately reconfigure their amplitude and phase response in time. For example, for a series of 100 chips used in a typical metasurface, the programming time would be approximately 100 µs, which also incorporates the time delay of the high-speed communication lines that connect the ASICs. Assuming there are four patches connected to each chip, this time can be translated into the time required to configure 400 metal-patches of a metasurface, each with individual settings. This ability is particularly useful, since modulation of arbitrary beam-shaping can be obtained, and thus the metasurface can control not only the propagation of electromagnetic energy within its environment, but also the information that the electromagnetic wave contains.

Programmable metasurfaces are expected to reciprocally co-evolve with telecommunication systems in the near future. Similarly, the ASICs presented are expected to evolve to satisfy the growing need for low-power and low-cost electronics tailored for programmable metasurfaces. The metasurface trend will require that future ASICs operate even faster and with wider programmable loading element ranges. Thus, technologies with smaller features are expected to be used in order to reduce the size of the chips and to increase their speed. Additionally, more exotic technologies that include memristors and/or micro-electromechanical switches will be utilized in the loading elements to increase their range. Furthermore, as silicon–germanium and gallium arsenide technologies become more affordable they will find their way into future ASICs for metasurfaces.

## Conclusions

We have developed a family of low-cost, low-power, high-speed scalable ASICs for complex impedance adaptation at microwave frequencies. The ASICs can communicate on metasurfaces and can be programmed via software to control the complex impedance of each loading element in time and in space. This can be translated into a variation of the amplitude and phase response of both the reflected and transmitted waves, for both TE and TM polarizations on a metasurface, due to the multi-bit resolution of the loading elements in each ASIC. Furthermore, each meta-atom can be individually addressed, without interfering with the incoming electromagnetic waves because of the asynchronous operation of the control circuit, which leads to low electromagnetic noise emissions. Even with these additional functionalities, each member of the ASIC family requires only microwatts for its static operation. The performance of the chips highlights the potential of using ASICs for realizing programmable metasurfaces. This work is a step towards the development of real-time programmable metasurfaces that can be integrated into indoor/outdoor telecommunication systems. It also paves the way for the development of metasurfaces for use in extraordinary applications that previously seemed infeasible, such as cloaking of not only static but also moving objects, moving holograms, dynamic manipulation of incoming signals and many more.

## Methods

### Chip fabrication and packaging

The dies were fabricated in a commercial 0.18 µm CMOS process technology. They were then retrieved by a third-party company for packaging using the Wafer Level Chip Scale Package (WLCSP) technology, forming each individual chip used in this work. This approach minimizes the parasitics added to the input nodes due to the wire bond connections of typical packages. The solder balls used are lead-free SAC405 solder spheres.

### PCB fabrication and population

The PCBs used for testing the chips were designed in-house and fabricated using a commercial PCB manufacturing technology. Each PCB board was populated with one chip. The use of via-in-pad technology was used to connect to the chip pads due to the very small pitch of the pads (0.4 µm). Each board had specific connections available to form an array of PCBs/chips, enabling the testing of various grid topologies. The PCBs used for the measurements, along with the de-embedding PCBs were designed on a Rogers 4350B substrate.

### Experimental setup

All measurements were performed at room temperature in a controlled environment with ESD protection. During the communication measurements, an OPAL KELLY XEM6010 module that uses a Xilinx Spartan-6 FPGA was used to serially sent bits to the device under test (DUT) board that has one ASIC soldered on it. To choose the number of bits to be sent and their values, a graphical user interface was developed. The GUI is provided in the Supplementary Information Note 3. The FPGA respects the dual-rail asynchronous communication protocol of the ASICs but it cannot follow the speed of the ASIC. To measure the maximum speed of the ASICs, we took the output of the ASIC and fed it back to the input while generating acknowledge as well. This way the ASIC was stuck in a loop with itself by sending and receiving its own packet at its maximum speed allowing us to measure it.

For power consumption measurements, a Keithley 4200 SCS was used to provide power to the chips and measure the current drawn by the system. To measure the energy consumption of the ASICs during switching activity, the same FPGA (OPAL KELLY XEM6010) was used. To visualize the signals exchanged in the channel between the FPGA and the DUT, a Tektronix MSO70604C mixed-signal oscilloscope was used. The energy consumption measurements were performed with the FPGA at a 2 MHz clock signal by continuously sending bits to the ASIC. The average current during switching activity is measured by the Keithley device and we performed the required calculations to convert it to Joules/bit.

The loading elements were measured using a Keysight N5227B four-port vector network analyzer (VNA). The VNA acquires the scattering parameters in Touchstone file format. A MATLAB^©^ script was used to control the VNA and simultaneously program the ASIC through the FPGA. The Touchstone files are de-embedded with the through-reflect-line technique.

## Supplementary Information

Supplementary Information 1.

Supplementary Information 2.

## Data Availability

All data generated or analyzed during this study are included in this published article and its supplementary information files.

## References

[1] Zhirihin D, Simovski C, Belov P, Glybovski S (2017). Mushroom high-impedance metasurfaces for perfect absorption at two angles of incidence. IEEE Antennas Wirel. Propagat. Lett..

[2] Badloe T, Mun J, Rho J (2017). Metasurfaces-based absorption and reflection control: perfect absorbers and reflectors. J. Nanomater..

[3] Kossifos KM, Antoniades MA, Georgiou J (2020). Integrated-circuit enabled adaptive metasurface absorber with independent tuning of orthogonal polarization planes. IEEE Access.

[4] Kossifos KM (2020). Toward the realization of a programmable metasurface absorber enabled by custom integrated circuit technology. IEEE Access.

[5] Pitilakis A (2020). A multi-functional reconfigurable metasurface: Electromagnetic design accounting for fabrication aspects. IEEE Trans. Antennas Propag..

[6] Liu F (2019). Intelligent metasurfaces with continuously tunable local surface impedance for multiple reconfigurable functions. Phys. Rev. Appl..

[7] Sun S (2012). High-efficiency broadband anomalous reflection by gradient meta-surfaces. Nano Lett..

[8] Shi H (2015). Gradient metasurface with both polarization-controlled directional surface wave coupling and anomalous reflection. IEEE Antennas Wirel. Propag. Lett..

[9] Wong AMH, Eleftheriades GV (2018). Perfect anomalous reflection with a bipartite huygens’ metasurface. Phys. Rev. X.

[10] Epstein A, Wong JPS, Eleftheriades GV (2016). Cavity-excited Huygens’ metasurface antennas for near-unity aperture illumination efficiency from arbitrarily large apertures. Nat. Commun..

[11] Mias C, Yap JH (2007). A varactor-tunable high impedance surface with a resistive-lumped-element biasing grid. IEEE Trans. Antennas Propag..

[12] Costa F, Monorchio A, Vastante GP (2011). Tunable high-impedance surface with a reduced number of varactors. IEEE Antennas Wirel. Propag. Lett..

[13] Komar A (2017). Electrically tunable all-dielectric optical metasurfaces based on liquid crystals. Appl. Phys. Lett..

[14] Li A (2017). High-power transistor-based tunable and switchable metasurface absorber. IEEE Trans. Microw. Theory Tech..

[15] Taravati S, Eleftheriades GV (2021). Programmable nonreciprocal meta-prism. Sci. Rep..

[16] Yang H, Yu T, Wang Q, Lei M (2017). Wave manipulation with magnetically tunable metasurfaces. Sci. Rep..

[17] Hu, F., Otter, W. J. & Lucyszyn, S. Optically tunable THz frequency metamaterial absorber. In *2015 40th International Conference on Infrared, Millimeter, and Terahertz waves (IRMMW-THz)* 1–2 (2015). 10.1109/IRMMW-THz.2015.7327423.

[18] Kossifos, K. M., Antoniades, M. A., Georgiou, J., Jaafar, A. H. & Kemp, N. T. An Optically-programmable absorbing metasurface. In *2018 IEEE International Symposium on Circuits and Systems (ISCAS)* 1–5 (IEEE, 2018). 10.1109/ISCAS.2018.8351874.

[19] Georgiou, J., Kossifos, K. M., Antoniades, M. A., Jaafar, A. H. & Kemp, N. T. Chua Mem-Components for Adaptive RF Metamaterials. In *2018 IEEE International Symposium on Circuits and Systems (ISCAS)* 1–5 (IEEE, 2018). 10.1109/ISCAS.2018.8351852.

[20] Yang H (2016). A programmable metasurface with dynamic polarization, scattering and focusing control. Sci. Rep..

[21] Chen K (2017). A reconfigurable active huygens’ metalens. Adv. Mater..

[22] Li L (2017). Electromagnetic reprogrammable coding-metasurface holograms. Nat. Commun..

[23] Zhao J (2019). Programmable time-domain digital-coding metasurface for non-linear harmonic manipulation and new wireless communication systems. Natl. Sci. Rev..

[24] Li Y, Shuang Y, Alù A (2019). Machine-learning reprogrammable metasurface imager. Nat. Commun..

[25] Zhang L (2018). Space-time-coding digital metasurfaces. Nat. Commun..

[26] Wang HL, Ma HF, Chen M, Sun S, Cui TJ (2021). A reconfigurable multifunctional metasurface for full-space controls of electromagnetic waves. Adv. Funct. Mater..

[27] Venkatesh S, Lu X, Saeidi H, Sengupta K (2020). A high-speed programmable and scalable terahertz holographic metasurface based on tiled CMOS chips. Nat. Electron..

[28] Liaskos C (2018). A new wireless communication paradigm through software-controlled metasurfaces. IEEE Commun. Mag..

[29] Tang W (2020). Wireless communications with programmable metasurface: new paradigms, opportunities, and challenges on transceiver design. IEEE Wirel. Commun..

[30] Zhao H (2020). Metasurface-assisted massive backscatter wireless communication with commodity Wi-Fi signals. Nat. Commun..

[31] Petrou, L., Karousios, P. & Georgiou, J. Asynchronous circuits as an enabler of scalable and programmable metasurfaces.

[32] Kossifos, K. M., Antoniades, M. A. & Georgiou, J. ASIC-Enabled Reprogrammable Metasurfaces for 5G Applications. In *2021 15th European Conference on Antennas and Propagation (EuCAP)* 1–4 (IEEE, 2021). 10.23919/EuCAP51087.2021.9411106.

[33] Kossifos, K. M., Antoniades, M. A. & Georgiou, J. Agile and multifunctional integrated-circuit-enabled metasurface. *2021 Int. Appl. Comput. Electromagn. Soc. Symp. ACES 2021* **2**, 6–9 (2021).

[34] Kossifos, K. M., Georgiou, J. & Antoniades, M. A. An IC-Enabled Metasurface Producing OAM and Pencil Beams. In *2021 IEEE International Symposium on Antennas and Propagation and USNC-URSI Radio Science Meeting (APS/URSI)* 299–300 (IEEE, 2021). 10.1109/APS/URSI47566.2021.9703945.

[35] Garrou P (2000). Wafer level chip scale packaging (WL-CSP): an overview. IEEE Trans. Adv. Packag..

[36] Amer AAG, Sapuan SZ, Nasimuddin N, Alphones A, Zinal NB (2020). A comprehensive review of metasurface structures suitable for RF energy harvesting. IEEE Access.

[37] *Principles of Asynchronous Circuit Design*. (Springer US, 2001). 10.1007/978-1-4757-3385-3.

[38] Amri MM, Tran NM, Choi KW (2021). Reconfigurable intelligent surface-aided wireless communications: adaptive beamforming and experimental validations. IEEE Access.

[39] Wu Z, Ra’di Y, Grbic A (2019). Tunable metasurfaces: a polarization rotator design. Phys. Rev. X.

