# Supplementary material for: The first family of application-specific integrated circuits for programmable and reconfigurable metasurfaces
Source: Sci Rep. 2022 Apr 6;12:5826. doi: 10.1038/s41598-022-09772-y (PMC8987096; doi:10.1038/s41598-022-09772-y)
Supplement: Supplementary file 2 — Supplementary Information 2. [file 41598_2022_9772_MOESM2_ESM.docx]

**The first family of application-specific integrated circuits for programmable and reconfigurable metasurfaces**

[
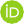
](https://orcid.org/0000-0002-9699-2387)[
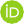
](https://orcid.org/0000-0002-7474-5449)[
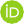
](https://orcid.org/0000-0002-2925-195X)[
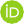
](https://orcid.org/0000-0003-1999-5733)Loukas Petrou 1✉*, Kypros M. Kossifos 1, Marco A. Antoniades 1,2 and Julius Georgiou 1

1Department of Electrical and Computer Engineering, University of Cyprus, Nicosia, Cyprus ✉e-mail: petrou.loukas@ucy.ac.cy;

^2^Department of Electrical, Computer and Biomedical Engineering, Ryerson University, Toronto, Canada

**Supplementary Information**

## Supplementary Note 1. Loading Element Measurements

The family of ASICs operates at the sub-6GHz band with four versions that are optimized for 5 GHz applications (Designs 1, 2, 5 and 6) and two designs for 3 GHz applications (Designs 3 and 4). The designs also possess two chip footprint versions. The chip footprint for Designs 1 and 2 can be seen in Figure 1(a). This footprint has a separate ground for the control circuit (DGND) and the RF ground (AGND). For these two designs an RF choke can be added in path of the RF ground of the ASIC and the PCB’s ground. This is particularly useful when there is a need to have a floating loading element. Designs 3 to 6 pin map, shown in Figure 1(b), have digital and RF common ground and each loading element has three ground pins surrounding the loading element for coupling protection and robust grounding. Table 1 shows the pin descriptions of the pins.


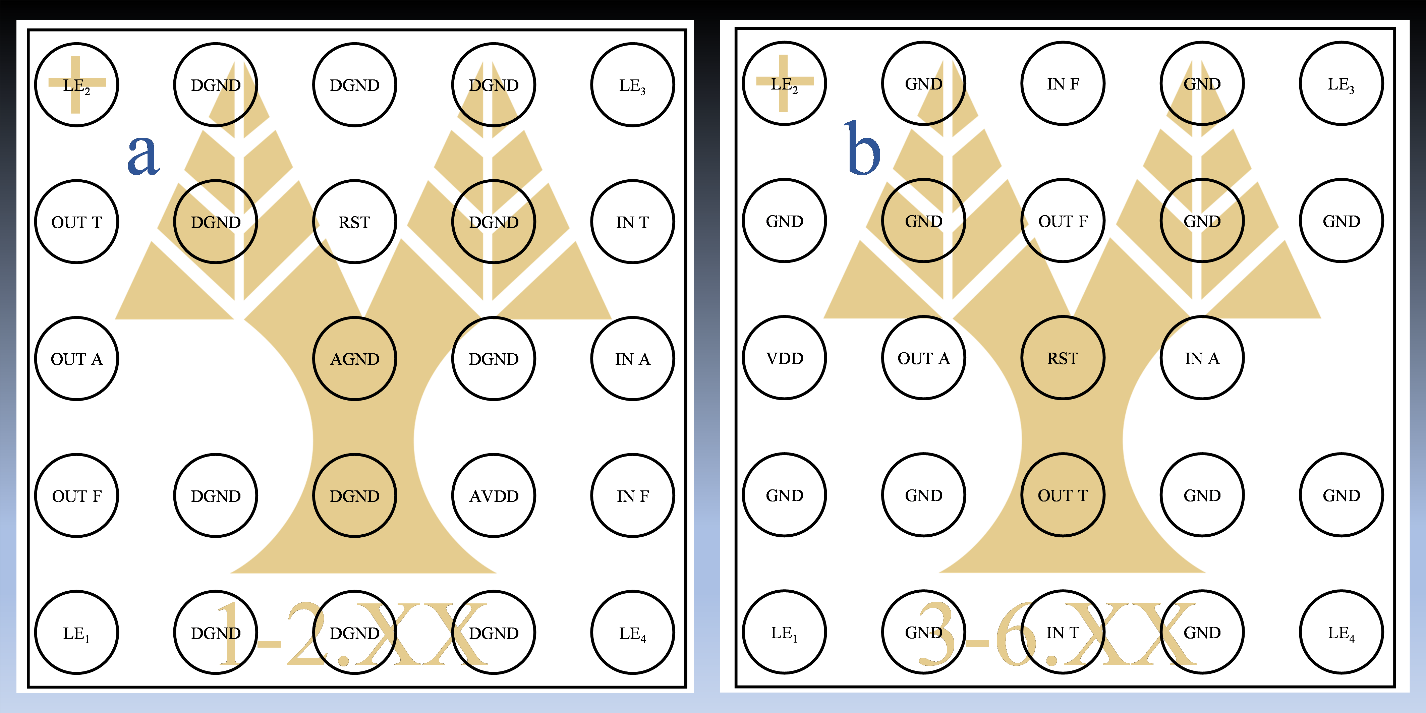


**Figure. 1 |** Chip footprints of the family of ASICs when looking at the solder spheres (bottom view). (a) Footprint for designs 1 and 2 and (b) Footprint for designs 3 to 6.

| Pin Name | Description |
| --- | --- |
| LE_x_ | Loading Element x terminal/port |
| IN T | Input True Signal |
| IN A | Input Ack Signal |
| IN F | Input False Signal |
| OUT T | Output True Signal |
| OUT A | Output Ack Signal |
| OUT F | Output False Signal |
| DVDD | Digital VDD |
| AVDD | Analogue VDD |
| DGND | Digital Ground |
| AGND | RF Ground |
| GND | Common Digital and RF Ground |
| RST | Negative Trigger Reset (or $\bar{\mathrm{RST}}$) |

**Table 1:** Pin Description

## Supplementary Note 2. 4-phase dual-rail protocol

The 4-phase dual-rail protocol is an asynchronous digital communication protocol for transmitting/receiving data without the use of external clock signal. Instead, it uses handshaking, using the well-known ‘request’ (dual-rail representation) and ‘acknowledge’ signal-wires. It is a robust protocol due to its delay insensitivity to gate-delays as opposed to other typical asynchronous communication protocols. In Figure 2, a graphical interpretation of the protocol is illustrated. Figure 2(a) shows the signals exchanged in the channel. We use two wires for every bit that we transmit thus the data bus requires 2n wires (n represents the number of bits that we transmit in parallel). One wire of a bit is the ‘data.true’ or ‘d.t’ and used for signal logic ‘1’ and the other wire is ‘data.false’ or ‘d.f’ and used for signal logic ‘0’. The request signal is encoded into the data signals and is set to ‘1’ only when all data signals are valid. A valid bit is considered when one of the two wires is logic ‘1’ as shown in the table of Figure 2(b). In Figure 2(c), the signal transitions in the channel is shown and the procedure is as follows:

Starting the communication, all signals are ‘0’ which means we have ‘Empty’ data and the ‘Ack’ signal is low. Then, the sender issues valid data as shown in Figure 2(b). The receiver stores the valid data and acknowledges this, through ‘Ack’ = ‘1’. The data then becomes ‘Empty’ and ‘Ack’ becomes ‘0’. At this point, the sender and the receiver have exchanged one bit of information and the next cycle is ready to begin. A simpler representation of the communication is shown in Figure 2(d), which only shows the state transition. Notice that whatever the data is, the state must return to ‘Empty’ before issuing new data. This is known as ‘return-to-zero’ or ‘RTZ’ line coding.

**Figure 2.** The 4-phase dual-rail protocol. (a) Communication channel between two consecutive nodes (b) Data representation (c), Signal transition for bit-to-bit communication and (d), State Diagram for bit-to-bit communication.

Figure 3 shows the timing diagram of the 4-phase dual-rail protocol. Time starts at the top of the diagram and moves downwards. The diagram assumes one bit is exchanged thus the total number of wires for the communication are three (data.t, data.f, and ack).


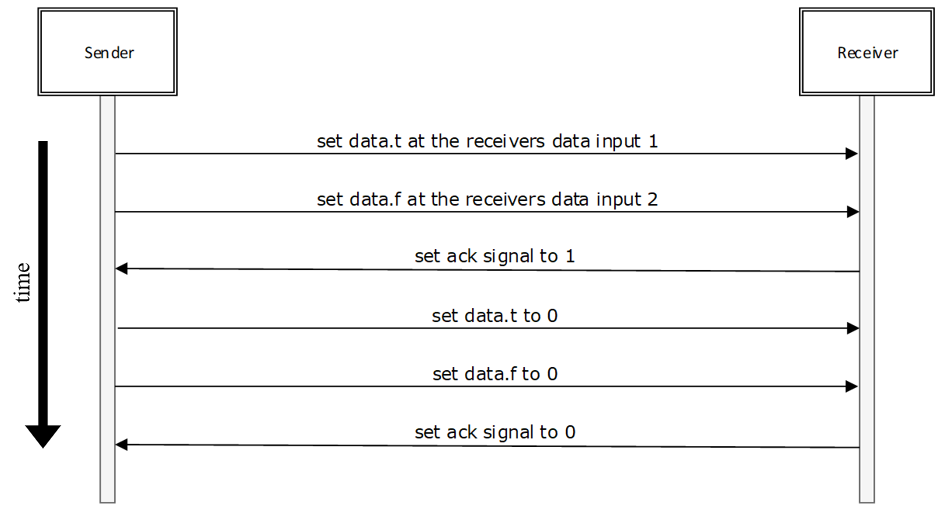


**Figure 3.** Timing diagram of one communication cycle between a sender node and a receiver node

## Supplementary Note 3. Graphical user Interface (GUI) for testing the communication performance.

Two programs were created for testing the chips, one for manual operation and one for automatic operation. For each of these, a graphical user interface (GUI) has been created to visualize the response of the chips.

### Manual Operation of Chip

The GUI for the manual operation is shown in Figure 4. Figure 4(a) shows the GUI, andFigure 4(b) shows part of the .xml file that creates the objects on the interface. For example, the text at the top of the GUI, ‘Buttons (input to the chip)’ is of type ‘StaticBox’ and in the .xml file its position, size, and label can be defined.

In this program, the user can manually set the value of each input (to the chip) signal. The output (of the chip) signals are set by the chip and the user can visualize these results. The user must manually execute the dual-rail protocol in order to communicate with the chip and send it packets. Below (Table 2), we describe each box of the GUI.

| **Buttons and Digits for the manual operation GUI.** | |
| --- | --- |
| **Buttons** | |
| Start | Start the program |
| Set VDD/GND | Make VDD value = ‘1’ and GND = ‘0’ |
| Set HRST | Make Hard Reset = ‘1’ (Negative Triggered) |
| Send bit ‘1’ | Make In-t value = ‘1’ and In-f value = ‘0’ |
| Send bit ‘0’ | Make In-f value = ‘1’ and In-t value = ‘0’ |
| Set out-a | Make Out-a value = ‘1’ |
| **Digits** | |
| In-a value | Acknowledgment value from chip |
| Out-t value | Output true value from chip |
| Out-f value | Output false value from chip |

**Table 2.** Buttons and Digits for the manual operation of the GUI.

| 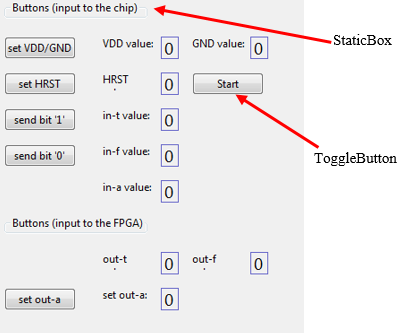 | 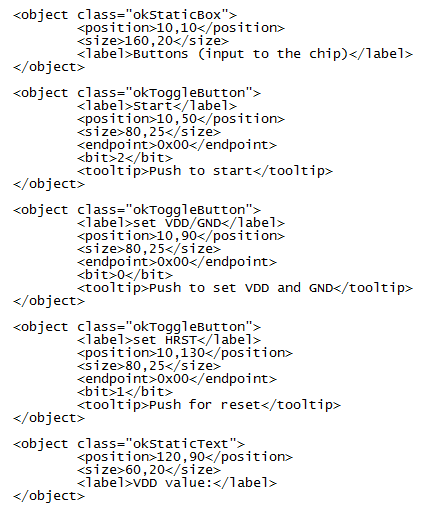 |
| --- | --- |
| (a) | (b) |

**Figure 4.** (a), Graphical User Interface for the manual program and (b), Xml file that created the objects in a.

### Automatic Operation of Chip

The automatic program respects the dual-rail protocol and sends multiple bits serially to the chips. Specifically, the program sends to the chip 128 bits that are chosen by the user in hexadecimal (HEX) form, entered through the GUI shown in Figure 5. At the beginning of the program, the user specifies the packet in the boxes under the ‘Set 128-bit of packet’ text. The bits are sent starting from the MSB and ending at the LSB. The program also keeps track of the bits sent (‘Bit counter’) and informs the user when the packet is successfully sent (‘End of packet’ flashes red).


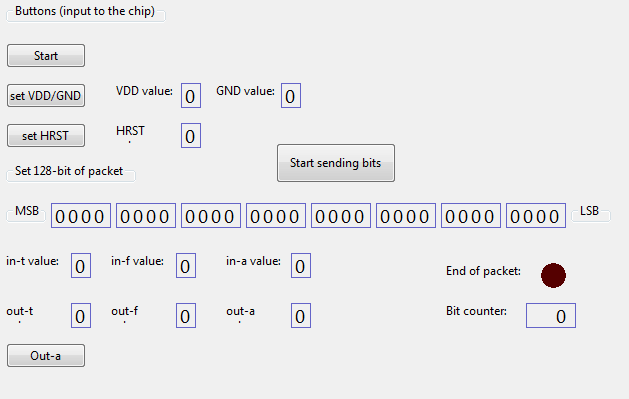


**Figure 5.** Graphical User Interface for the Automatic Operation of the Chip
